# The binding of the small heat-shock protein αB-crystallin to fibrils of α-synuclein is driven by entropic forces

**DOI:** 10.1073/pnas.2108790118

**Published:** 2021-09-13

**Authors:** Tom Scheidt, Jacqueline A. Carozza, Carl C. Kolbe, Francesco A. Aprile, Olga Tkachenko, Mathias M. J. Bellaiche, Georg Meisl, Quentin A. E. Peter, Therese W. Herling, Samuel Ness, Marta Castellana-Cruz, Justin L. P. Benesch, Michele Vendruscolo, Christopher M. Dobson, Paolo Arosio, Tuomas P. J. Knowles

**Affiliations:** ^a^Centre for Misfolding Diseases, Yusuf Hamied Department of Chemistry, University of Cambridge, Cambridge CB2 1EW, United Kingdom;; ^b^Department of Chemistry, Physical & Theoretical Chemistry, Chemical Research Laboratory, University of Oxford, Oxford OX1 3TA, United Kingdom;; ^c^Department of Chemistry and Applied Biosciences, ETH Zurich, 8093 Zurich, Switzerland;; ^d^Cavendish Laboratory, Department of Physics, University of Cambridge, Cambridge CB3 0HE, United Kingdom

**Keywords:** microfluidics, aggregation, chaperones, thermodynamic, kinetic analysis

## Abstract

Molecular chaperones are key components of the cellular proteostasis network whose role includes the suppression of the formation and proliferation of pathogenic aggregates associated with neurodegenerative diseases. The molecular principles that allow chaperones to recognize misfolded and aggregated proteins remain, however, incompletely understood. To address this challenge, here we probe the thermodynamics and kinetics of the interactions between chaperones and protein aggregates under native solution conditions using a microfluidic platform. We focus on the binding between amyloid fibrils of α-synuclein, associated with Parkinson’s disease, to the small heat-shock protein αB-crystallin, a chaperone widely involved in the cellular stress response. We find that αB-crystallin binds to α-synuclein fibrils with high nanomolar affinity and that the binding is driven by entropy rather than enthalpy. Measurements of the change in heat capacity indicate significant entropic gain originates from the disassembly of the oligomeric chaperones that function as an entropic buffer system. These results shed light on the functional roles of chaperone oligomerization and show that chaperones are stored as inactive complexes which are capable of releasing active subunits to target aberrant misfolded species.

## Significance

The formation of amyloid fibrils and toxic oligomeric species has been shown to be inhibited by their interactions with molecular chaperones, thus modulating monomer sequestration and toxicity in the context of neurodegenerative diseases. Understanding the physical and chemical properties underlying chaperone binding processes is essential to explore new therapeutic strategies to target toxic amyloid species. Here, we determine that the binding of the small heat-shock protein αB-crystallin to α-synculein fibrils, a protein which is related to the progression of Parkinson’s disease, is driven by entropic forces. By applying a microfluidic platform, we accurately quantified the thermodynamics and the kinetics of this intermolecular interaction in the condensed phase and hypothesize that αB-crystallin oligomers work as an entropic buffer system.

Molecular chaperones are crucial components of the cellular proteostasis network and are characteristically overexpressed during cell stress (1–4). Their roles involve the suppression of aberrant processes, including misfolding and aggregation of proteins, within the context of the complex flux of protein production and degradation. In addition to guiding nascent proteins toward their native structures following biosynthesis on ribosomes, chaperones are increasingly recognized as inhibitors of key steps in the aberrant conversion of normally soluble proteins into amyloid fibrils, protein aggregates that are associated with a wide range of neurodegenerative diseases (5–8). The overall process that leads to the formation of amyloid fibrils consists of a series of microscopic events, including primary and secondary nucleation and fibril elongation and fragmentation (9). Recent analysis of the kinetics of aggregation of several proteins has revealed that molecular chaperones can inhibit the process of amyloid formation through a variety of different microscopic mechanisms (10). In some cases, molecular chaperones have been found to suppress a single specific microscopic step in the aggregation process. In other cases, they have been shown to affect more than one type of aggregation event (7, 8, 11, 12). The modulation of the different molecular steps of protein aggregation is mediated by the binding of chaperones to misfolded protein monomers and various aggregates (11, 13, 14). For a comprehensive understanding of such inhibition processes it is therefore crucial to elucidate the thermodynamic and kinetic determinants of the binding of chaperones to different species populated during amyloid formation.

A prevalent group of molecular chaperones that inhibit amyloid formation are the small heat-shock proteins (sHsps), including the vertebrate αB-crystallin (αB-c). The structure of αB-c is a conserved α-crystallin domain with a β-sheet structure, flanked by a hydrophobic N-terminal region and a polar C-terminal tail, both structurally flexible and mutually different (15). Similar to other sHsps in solution, αB-c exists in a polydisperse oligomeric state characterized by dynamic subunit exchange leading to oligomers with 10 to 50 subunits and molecular weights from 300 to 1,000 kDa (16, 17). αB-c has been shown to inhibit the overall amyloid formation process of α-synuclein (α-syn), a protein closely associated with the onset and progression of Parkinson’s disease (18). The mechanism of inhibition has been shown to originate from interactions of the chaperone with aggregated forms of α-syn, ranging from oligomers to mature amyloid fibrils, rather than with α-syn monomers (19, 20). In particular, it has been demonstrated that αB-c binds to α-syn fibrils and inhibits their elongation in solution, thus suppressing the toxicity associated with α-syn aggregation in cells (13, 21).

The mechanistic importance of the interactions of αB-c with protein aggregates raises the key question of how chaperones recognize misfolded and aggregated proteins among the diverse ensemble of native states. Elucidating the binding interactions between these proteins poses fundamental challenges that originate from the heterogeneity and dynamic nature of the systems. Both the chaperone and aggregate populations are polydisperse, and the large difference in size between relatively small chaperones and high-molecular-weight client protein aggregates make interactions between them difficult to access with conventional biophysical techniques designed to probe interactions between individual biomolecules (22, 23). We have addressed these limitations using a microfluidic platform to characterize the binding (24). By exploiting the different diffusion coefficients of bound and unbound chaperones we have shown that it is possible to quantify the thermodynamics and the kinetics of binding on the time scale of minutes, where the spatial variation in concentration along the device has a negligible effect on the kinetics due to the short measurement times (24, 25). Here, we apply this approach to identify the intermolecular interactions underlying the recognition of α-syn amyloid fibrils by αB-c and to characterize the energetic trade-off during this binding process in a quantitative manner.

## Results

### Microfluidic Measurements of Protein Interactions in the Condensed Phase.

**Fig. 1.**
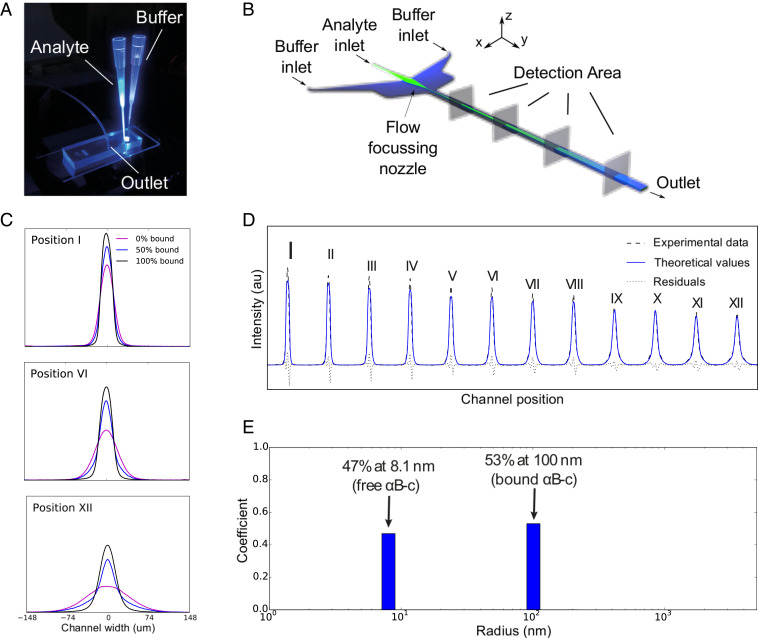
(*A*) Image of the microfluidic diffusion device on an epifluorescence microscope. Pipette tips hold the analyte and buffer, and flow is controlled by applying negative pressure at the outlet. (*B*) Schematic of the device. The analyte is focused between two buffer streams, and diffusion profiles are recorded at discrete detection points along the channel. Reproduced with permission from ref. 24 (Copyright 2016, American Chemical Society). (*C*) Experimental profiles of three chaperone-fibril samples exhibit different extent of binding: 0% (purple), about 50% (blue), and 100% (black) of total chaperones bound to fibrils. Normalized profiles are shown for three channel positions. The sample with 0% bound chaperone diffuses the most, whereas the sample with 100% bound chaperone stays most localized in the center of the channel. Partially bound samples exhibit superposition of two peak shapes: diffuse (corresponding to free chaperone) and localized (corresponding to bound chaperone). (*D*) Experimental profiles of αB-c (1 μM) binding to α-syn fibrils (10 μM) at 25°C at 12 diffusion positions along the channel and the simulated profiles for the 50% bound sample. (*E*) The fitting of the model simulations to the experimental data provides a direct measure of the fraction of particles in each size bin.

The microfluidic diffusional sizing (MDS) approach allows us to determine the hydrodynamic radii ($R_{H}$) of individual components of a complex mixture of species and to quantify their relative concentrations. Briefly, the approach consists of acquiring quantitative data on micrometer-scale mass transport in two dimensions, both time and space, and deconvolving these global profiles into the contribution of the individual components within the mixture, thus yielding the distribution of diffusion coefficients (24). This approach makes use of laminar flow within a microfluidic channel (Fig. 1 *A* and *B*) both to create a well-defined interface between the analyte and a running buffer and to control the diffusion time. The diffusion coefficients are then directly related to hydrodynamic radii via the Stokes–Einstein equation, $D=\left(k_{B}T\right)/\left(6\pi \eta R_{H}\right)$, where *D* is the diffusion coefficient, *T* is the temperature, *k*_*B*_ is the Boltzmann constant, and η is the solvent viscosity.

First, the diffusion profiles of green fluorescent protein (GFP)-labeled αB-c were acquired by epifluorescence microscopy in the absence and presence of unlabeled α-syn fibrils. By exploiting the difference in diffusion coefficient between amyloid fibrils and free molecular chaperones in solution we can detect changes in the size distribution upon the formation of the complex between the chaperone αB-c and amyloid fibrils (Fig. 1 *C* and *E*). When a labeled chaperone binds to a fibril, the complex formed exhibits a diffusion coefficient about one order of magnitude smaller than the diffusion coefficient of a free chaperone. This difference allows us to deconvolve the diffusion profiles into the relative contribution of the two components, the rapidly diffusing free chaperones and the slowly diffusing chaperone–fibril complexes, and this determines in free solution the absolute concentrations of both binding partners.

Before measuring the binding parameters, we verified by means of kinetic analysis that the GFP tag on the αB-c does not affect the chaperone’s capability to inhibit α-syn amyloid formation. To do so, we followed the aggregation kinetics of a solution of 70 μM α-syn to which 5% wt/wt preformed seeds had been added in the presence and absence of different concentrations of GFP itself, and both unlabeled and labeled αB-c (*SI Appendix*, Fig. S1). We observed very similar aggregation profiles for the two chaperone types, indicating that the GFP tag does not significantly modify the binding properties of αB-c to α-syn fibrils and its ability to inhibit their aggregation. Furthermore, no effect on α-syn aggregation by the presence of GFP itself was detected. This indicates there are no interactions between GFP and aggregation-relevant binding sites of α-syn.

By using our microfluidic approach we measured the average hydrodynamic radius, $R_{H}$, of the polydisperse αB-c oligomer distribution to be 8.7 ± 0.5 nm at 21^○^C (*SI Appendix*, Fig. S2). This value is in good agreement with previously reported values measured by size-exclusion chromatography and dynamic light scattering ($R_{H}$ = 7.25 nm), with the attached GFP moiety being responsible for the increased measured size relative to the wild type (26). The measured $R_{H}$ indicates that αB-c is present as an ensemble of oligomers, which is in agreement with mass spectrometric analysis (*SI Appendix*, Fig. S3). Similar to native mass spectrometry measurements, our microfluidic measurements indicate there are, if any, very few or transient interactions between the αB-c complex and α-syn monomers (*SI Appendix*, Fig. S4) (13). When α-syn fibrils were introduced in the system and the distribution of hydrodynamic radii was measured using the microfluidic platform, a second size of species in the $R_{H}$ distribution emerged in the range of 70 to 150 nm (Fig. 1). This second population, with a larger radius, corresponds to the complex formed by αB-c and α-syn fibrils and thus reports on the interactions between these two species.

### Experiments under Native Conditions Reveal a Nanomolar Affinity for αB-c Binding to α-syn Fibrils.

**Fig. 2.**
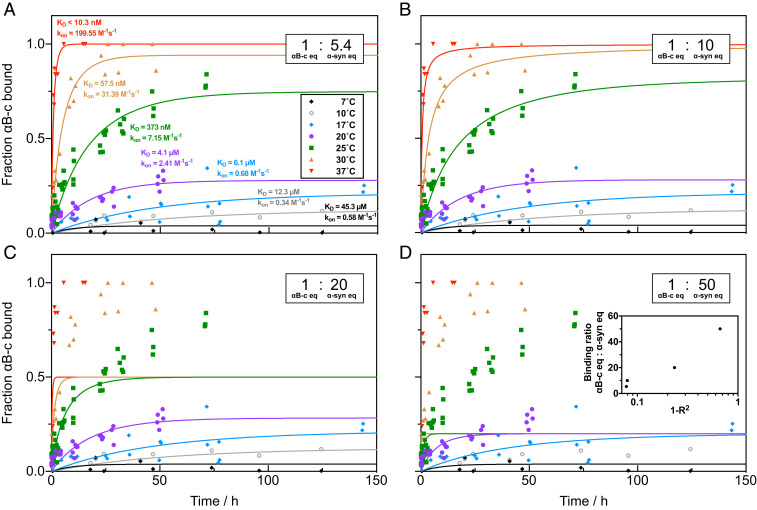
Kinetic data of αB-c (1 μM) binding to α-syn fibrils (10 μM) reveal kinetic parameters of binding. Kinetic traces at different temperatures were fit to a second-order rate equation to obtain the association ($k_{on}$) and dissociation ($k_{o\mathrm{ff}}$) rate constants, from which an apparent dissociation constant $K_{D,app}$ was calculated. Different binding ratios of αB-c equivalents (eq) to α-syn fibril mass eq with (*A*) 1:5.4, (*B*) 1:10, (*C*) 1:20, and (*D*) 1:50 were simulated. The resulting ${1-R}^{2}$ values are plotted against the corresponding binding ratios as an inset in *D*. The optimal fit (*A*) is given with a stoichiometry of 1:5.4 with a lower boundary for the stoichiometry at 1:8 given by the SD.

We next exploited the quantitative power of the technique to evaluate the affinity of the chaperone–fibril interactions in the condensed phase under native conditions. To this effect, we characterized the kinetics of the binding reaction by incubating a solution of 1 μM αB-c with a suspension of 10 μM α-syn fibrils and measuring the size distribution of aliquots taken at discrete time points over the course of 50 to 150 h. In a wide range of protein concentrations the oligomer distribution of αB-c has been shown to be rather narrow and defined (26). We carried out the measurements at seven different temperatures (7°C, 10°C, 17°C, 20°C, 25°C, 30°C, and 37°C) (Fig. 2) to obtain both the thermodynamic parameters and the activation free energy involved in this binding process. In order to analyze quantitatively the binding kinetics at individual temperatures, we fitted the binding-site concentration globally as its value should be identical under all seven conditions tested.

Our kinetic data reveal that the reaction is first-order (Fig. 2*A*) with respect to both chaperone and fibril, and hence second-order overall. We obtained values for the effective association rate constants (e.g., $k_{on,25^{○}C}=7.1\pm 2.6\;{\text{M}}^{-1}\cdot {\text{s}}^{-1}$ and dissociation rate constants (e.g., $k_{{o\mathrm{ff},25}^{○}C}=2.7\times 10^{-6}\pm 8.6\times 10^{-7}$ $s^{-1}$). The accuracy of the fitted dissociation rates has been tested separately by following the dissociation of bound αB-c in a saturated α-syn fibril solution at 7°C (*SI Appendix*, Fig. S6). The dissociation rate constant $k_{{o\mathrm{ff},7}^{○}C}=5.6\times 10^{-5}\pm 1.2\times 10^{-5}$ $s^{-1}$ measured with this experiment is similar to the dissociation rate calculated from the association kinetics $k_{{o\mathrm{ff},7}^{○}C}=2.6\times 10^{-5}\pm 1.6\times 10^{-5}$ $s^{-1}$. The effective dissociation equilibrium constants are computed from the ratio of the rate constants (e.g., $K_{{D,app,25}^{○}C}=373\pm 238$ nM and $K_{D,app,20^{○}C}=4.1\pm 1.7$ μM). As the affinities are calculated with respect to the monomer equivalent concentration of αB-c, the given dissociation constants might represent an upper bound, because the binding species may in fact be monomeric, and because the species capable of binding may constitute only a fraction of the total αB-c concentration. Nevertheless, similar affinities have been reported for αB-c binding to other amyloid fibrils, including Aβ42 fibrils ($K_{D}=2.1$ μM), A$\beta 42_{arc}$ mutant fibrils ($K_{D}=0.34$ μM) (14), and apoC-II fibrils ($K_{D}=5.4$ μM) (27). Using the MDS platform, it is possible to elucidate the number of binding sites on a fibril. To do so, we examined the global influence of the binding stoichiometry on the binding kinetics by comparing the model predictions for different binding ratios of αB-c to α-syn between 1:5.4 and 1:50 (Fig. 2 *A*–*D*). The modeled data do not match the measured data points if the binding ratio of α-syn equivalents to αB-c equivalents increases beyond 8 (Fig. 2 *B* and *C*). This best fit gives one αB-c equivalent binding on average every 5.4 α-syn equivalents. Based on previous findings reporting αB-c as an inhibitor for fibril elongation, our evaluated stoichiometry indicates that αB-c not only binds to fibrillar ends but also interacts with fibrils in a multimeric state, along the fibril surface not interfering with secondary nucleation sites or both. Similar inhibition and binding behavior has been observed for other sHSP and amyloid systems, such as clusterin together with Aβ42 fibrils (8). At the highest temperature ($37^{○}C$) the association was fast and the fraction bound was (within error) 100%, hence yielding only an upper bound on $K_{D}$. For this reason the $K_{D}$ measured at $37^{○}C$ was not used for any further analysis.

In order to probe whether the binding reaction could be described as a two-state process we next sought to measure the value of the apparent equilibrium dissociation constant directly from equilibrium titration measurements and compared the value obtained to that of the estimated affinities given by the kinetic analysis. Concentrations of α-syn fibrils between 1 and 100 μM were incubated with 1 μM αB-c at 25°C for 3 d to ensure that the binding reaction had reached equilibrium (*SI Appendix*, Fig. S5). A noncooperative, single-site binding model (see *Materials and Methods*) was found to describe the titration data, using the previously calculated α-syn binding-site ratio of 1:5.4 as a fixed input parameter (Fig. 2*A*). The apparent dissociation constant given by the titration experiment is $K_{{D,app,25}^{○}C}=261\pm 76$ nM (*SI Appendix*, Fig. S5), which is in agreement with the $K_{D}$ obtained from the kinetic experiments at the same temperature, showing that both experiments probe the same thermodynamic landscape.

### The Binding of αB-c to α-syn Fibrils Exhibits Strong Entropy/Enthalpy Compensation.

**Fig. 3.**
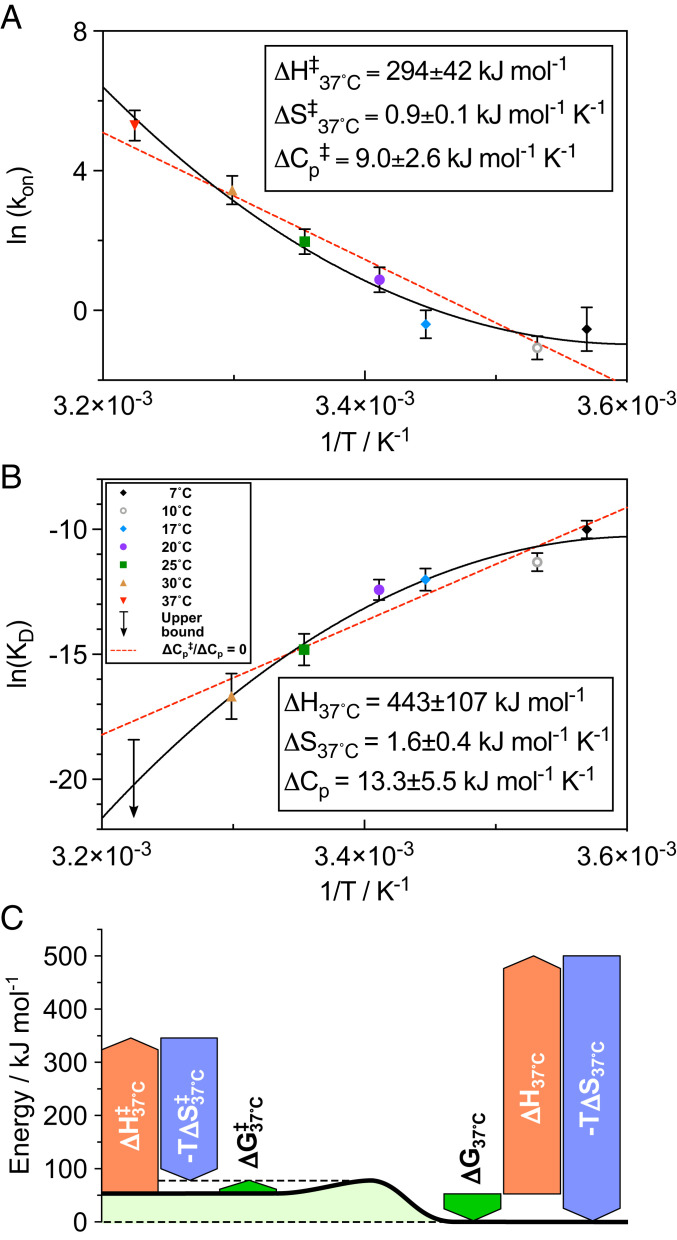
Thermodynamic parameters of αB-c (1 μM) binding to α-syn fibrils (10 μM) derived from the kinetic parameters of binding. (*A*) The enthalpic ($\Delta H_{37^{○}C}^{‡}=294\pm 42\;\text{kJ}\cdot {\text{mol}}^{-1}$) and entropic ($\Delta S_{37^{○}C}^{‡}=0.9\pm 0.1\;\text{kJ}\cdot {\text{mol}}^{-1}\cdot {\text{K}}^{-1}$) contribution together with the change in heat capacity ($\Delta C_{p}^{‡}=9\pm 2.6\;\text{kJ}\cdot {\text{mol}}^{-1}\cdot {\text{K}}^{-1}$) involved in the formation of the activated state of the binding partners were estimated using a model which combines polymer theory and Kramer’s problem of escape from a metastable state. Therefore, the free energy barrier of binding is $\Delta G_{37^{○}C}^{‡}=24\pm 61\;\text{kJ}\cdot {\text{mol}}^{-1}$. The large barrier suggests the binding is a highly activated process. (*B*) Values of $K_{D,app}$ were plotted according to the van’t Hoff equation to obtain the binding enthalpy ($\Delta H_{37^{○}C}=443\pm 107\;\text{kJ}\cdot {\text{mol}}^{-1}$) and entropy ($\Delta S_{37^{○}C}=1.6\pm 0.4\;\text{kJ}\cdot {\text{mol}}^{-1}\cdot {\text{K}}^{-1}$) together with the change in heat capacity ($\Delta C_{p}=13.3\pm 5.5\;\text{kJ}\cdot {\text{mol}}^{-1}\cdot {\text{K}}^{-1}$). The binding is enthalpically unfavorable and entropically favorable. Value at 37 °C was not included in analysis and is only given as an upper bound. The dashed red lines in *A* and *B* show similar fits with $\Delta C_{p}^{‡}and\Delta C_{p}=0$, indicating that the change in heat capacity is positive. (*C*) The reaction diagram shows the fraction of the individual thermodynamic parameters and shows that the overall chaperone–fibril binding is spontaneous with a free energy of $\Delta G_{37^{○}C}=-52\pm 154\;\text{kJ}\cdot {\text{mol}}^{-1}$.

**Fig. 4.**
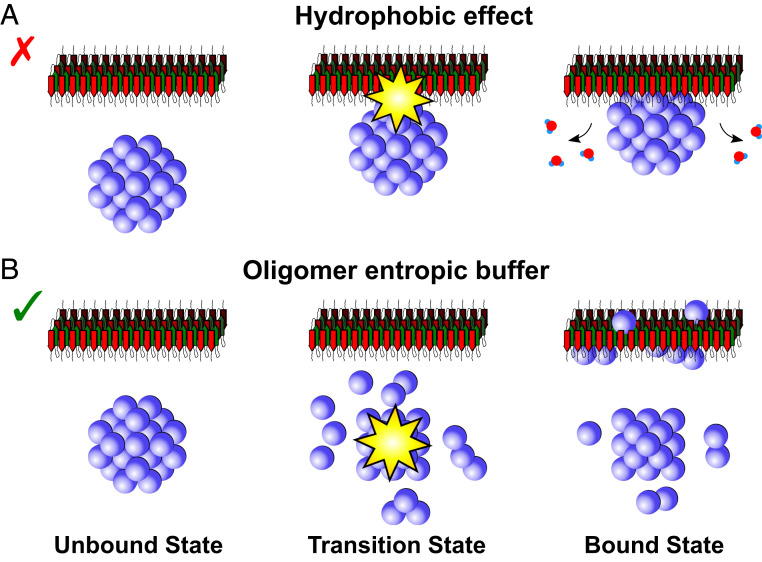
Binding mechanisms of αB-c to α-syn fibrils. The gain of entropy during the binding reaction of αB-c to α-syn fibrils can be explained either by (*A*) a solvent-mediated interaction through release of water molecules or (*B*) a conformational change of the binding partners. The observed positive change in heat capacity ($\Delta C_{p}$) supports the latter explanation.

The apparent dissociation constants estimated at different temperatures allowed us to deconvolve the enthalpic and entropic components of the free energy of binding using a nonlinear van’t Hoff analysis (Fig. 3*B*). The values obtained indicate that the binding between αB-c and α-syn fibrils is endothermic at 37°C ($\Delta H_{37^{○}C}=443\pm 107\;\text{kJ}\cdot {\text{mol}}^{-1}$), and that the enthalpic loss upon binding is compensated by a gain in entropy ($\Delta S_{37^{○}C}=1.6\pm 0.4\;\text{kJ}\cdot {\text{mol}}^{-1}\cdot {\text{K}}^{-1}$), resulting in an overall spontaneous process at physiological temperature ($\Delta G_{37^{○}C}=-52\pm 154\;\text{kJ}\cdot {\text{mol}}^{-1}$) (Fig. 3*C*). The gain in entropic energy that drives the binding of the chaperone to the surface can originate from two processes, illustrated in Fig. 4. A first possibility involves the reduction of the translational and rotational degrees of freedom of the proteins upon binding to the fibrils. In this case, the increase in entropy originates from the release of constraints on hydrogen bonding of water molecules resulting from the burial of hydrophobic protein patches upon binding or other solvent-mediated interactions (Fig. 4*A*). The release of water molecules and therefore an overall increase in entropy is typically the signature of binding driven by hydrophobic interactions. A second explanation of the entropic-driven binding reaction is the increase of degrees of freedom induced by conformational changes of the interacting molecules. In this case, the entropic gain would mainly originate from the disassembly of the oligomeric chaperones (Fig. 4*B*), while the interactions between fibrils and chaperones would be specific and mediated by the surface chemistry of the two binding partners. To discriminate between these two possibilities we measured the associated change in heat capacity (see *Materials and Methods*). We determined a positive change in heat capacity ($\Delta C_{p}=13.3\pm 5.5\;\text{kJ}\cdot {\text{mol}}^{-1}\cdot {\text{K}}^{-1}$), indicating that the entropic-driven binding is due to the disassembly of the chaperone oligomers into smaller subunits, rather than to a hydrophobic effect (Fig. 4*B*). This is supported by previous findings showing that the exchange of αB-c subunits occurs on a similar timescale as the binding between αB-c and α-syn fibrils (16). In contrast, the release of strongly coordinated water molecules from hydrophobic patches into bulk solution (Fig. 4*A*) would lead to a negative change of the heat capacity (28). We note that with the current set of data we cannot determine whether the overall reaction involves one single intermediate state or consists of multiple reaction steps. Therefore, the measured parameters refer to an apparent intermediate state. Overall, our results indicate the presence of a chaperone activation step through substrate/temperature-dependent disassembly of chaperone complexes. This mechanism is known for Hsp27 and is also consistent with previous findings on substrate activated and thermosensitive disassembly of other sHsp associated with the inhibition of protein aggregation (29, 30).

### Analysis of Thermodynamic Contributions to the Activated State.

The apparent association rate constant ($k_{on}$) measured here increases with temperature. These findings provide a thermodynamic explanation for the reported greater efficiency of αB-c in inhibiting α-syn aggregation at higher temperatures (20). From the association rate of αB-c to the fibrils as a function of temperature we estimated a free energy barrier of $\Delta G_{37^{○}C}^{‡}=24\pm {61\;\text{kJ}\cdot \text{mol}}^{-1}$ (Fig. 3*C*). Moreover, we measured the individual enthalpic ($\Delta H_{37^{○}C}^{‡}=294\pm 42\;\text{kJ}\cdot {\text{mol}}^{-1}$) and entropic ($\Delta S_{37^{○}C}^{‡}=0.9\pm 0.1\;\text{kJ}\cdot {\text{mol}}^{-1}\;K^{-1}$) contributions at 37°C as well as the change in heat capacity ($\Delta C_{p}^{‡}=9\pm 2.6\;\text{kJ}\cdot {\text{mol}}^{-1}\cdot {\text{K}}^{-1}$) necessary to reach this activated state (Fig. 3*A*) (see *Materials and Methods*) (31, 32). The observed high free energy barrier can be part of multiple intermediate steps and be related to αB-c conformational changes, including changes in the oligomeric state upon binding to the surface of the fibrils. Crucially, these results show that interactions between chaperones and amyloid fibrils are highly regulated and specific, which is reflected in the high free energy barriers.

## Discussion and Conclusion

The quantification of the thermodynamic and the kinetic parameters associated with the binding of αB-c to α-syn fibrils provides important information on the mechanisms through which molecular chaperones are able to recognize misfolded protein aggregates and interfere with their proliferation. The client-binding region of αB-c is still unconfirmed, although it has been hypothesized to involve the N-terminal domain and the conserved αB-c domain (23, 33–37). In fact, different residues of the chaperone may be relevant in binding to different clients. Recent studies have shown that αB-c is a potent inhibitor in cells and that the αB-c core domain inhibits the aggregation of α-syn with similar efficacy to the wild-type (full-length) protein in vitro (38, 39). Furthermore, it has been shown that αB-c binds to an amorphously aggregating client protein (reduced lysozyme) via its unstructured N-terminal domain, but interactions with an amyloid aggregating client (amyloid-$\beta_{1-40}$) were mediated by the structured α-crystallin domain (23, 40). Both areas are predicted to be hydrophobic and are buried in the oligomeric state of the protein. Our findings provide direct thermodynamic evidence that the recognition of amyloid fibrillar structures by αB-c is driven by entropic forces, which include disassembly of the chaperone and local structural ordering and disordering upon binding. The latter can be described as an “entropy transfer” model, where entropic costs of binding are paid for by entropy-increasing conformational changes within the protein (41, 42). Moreover, the high activation barrier associated with the binding process for αB-c supports the hypothesis that some structural or conformational rearrangements are necessary for binding to occur. This “entropic buffering” allow sHsps to cluster at normal conditions but shed components entropically with increasing temperature in order to inactivate (buffer) aberrant protein conformations.

An intriguing possible consequence of the interactions between chaperones and amyloid fibrils in living systems is the sequestration of chaperones by amyloid fibrils, with subsequent loss of function and development of toxicity. Sequestration and reduced activity of chaperones is particularly dangerous since it can have a sequential effect, due to the reduced ability of the cell to cope with any subsequent misfolding and aggregation (43). By contrast, a favorable effect of the binding of αB-c to α-syn fibrils is that the surface coverage by the chaperone may hinder α-syn secondary nucleation, which is consistent with imaging results of the binding of the closely related sHSP, HSP27, to α-syn (44).

Chaperones have a remarkable ability to bind a wide variety of misfolded substrate proteins. Our current thermodynamic characterization suggests that chaperone oligomers can bind to unfolded or misfolded proteins by conformational changes, in particular chaperone disassembly. The generic chaperone binding to misfolded protein surfaces paradigm might be facilitated by a repertoire of various chaperone conformations/oligomeric states and seem to be an ideal natural adaptation strategy for chaperones to detect diverse aggregation-prone species. Quantifying chaperone–amyloid interactions, along with dissection of amyloid aggregation pathways, will enhance our understanding of the possible mechanisms of inhibition and open attractive strategies for rational design of potential drug molecules which mimic these effects.

## Materials and Methods

### Proteins.

GFP-labeled αB-c was prepared by purifying a construct with the following arrangement: αB-c-TEV site-GFP-His tag. This construct was expressed recombinantly in *Escherichia coli* BL 21 (DE3). Cells were lysed using a microfluidizer and centrifuged to remove insoluble material and the fusion protein was isolated using Ni affinity chromatography, HisTrap column (GE Healthcare) using standard procedures. The protein was further purified by size-exclusion chromatography on a Superdex 200 16/60 column (GE Healthcare). The resultant protein was in a solution of 300 mM NaCl and 50 mM Tris (pH 8) made up in 20% glycerol (aq).

α-Syn was expressed and purified in phosphate-buffered saline (PBS), pH 7.2, as described previously (45, 46). For the Alexa 488 labeling a cystein variant (N122C) was used. The labeling protocol included incubation of the protein with an excess of Alexa 488 dye with maleimide moieties (Thermo Fisher Scientific) (overnight at 4°C) at a molar ratio of 1:1.5 (protein:dye). The labeling mixture was loaded onto a Superdex 200 16/600 (GE Healthcare) and eluted in PBS or 10 mM sodium phosphate buffer, pH 7.4, at 20°C, to separate the labeled protein from free dye. The concentration of the labeled protein was estimated by the absorbance of the fluorophores, assuming a 1:1 labeling stoichiometry (Alexa 488: 72,000 M^−1^⋅cm^−1^ at 495 nm).

To prepare fibrils of unlabeled α-syn, a solution of 70 μM monomeric α-syn and 0.1% NaN_3_ in PBS, pH 7.2, was incubated in an Eppendorf tube at 37°C under constant shaking at 200 rpm for 5 d. The visibly cloudy sample was centrifuged at 15,000 rpm for 5 min and the pellet was washed once with PBS, pH 7.2, with 0.1$\%\;{NaN}_{3}$ to remove residual monomer. The fibrils were resuspended at 100 μM and sonicated with the probe sonicator SONOPULS HD 2070 (BANDELIN electronic) at 10% power, 30% cycles for 1 min. Fibril concentration (measured in constituent monomer concentration) was measured by denaturing a fibril aliquot in 5.5 M GuHCl and measuring the resultant α-syn monomer absorbance. All chemicals were of analytical grade and purchased from Sigma-Aldrich unless otherwise stated.

### Fabrication and Use of Microfluidic Diffusion Devices.

The fabrication and the operation of the microfluidic diffusion device used in the present studies have been described in previous papers (24, 47). Briefly, the microfluidic chips were fabricated by using standard soft lithography. The sample to be analyzed and the buffer were introduced into the system through reservoirs connected to the inlets, and the flow rate in the channel was controlled by applying a negative pressure at the outlet by a syringe pump (Cetoni neMESYS) at typical flow rates in the range from 90 μL/h to 150 μL/h. Lateral diffusion profiles were recorded at 12 different positions (3.5, 5.3, 8.6, 10.3, 18.6, 20.3, 28.6, 30.4, 58.7, 60.4, 88.7, and 90.5 mm) by standard epifluorescence microscopy using a cooled charge-coupled device camera (Photometrics Evolve 512). The diffusion profiles were fitted to model simulations based on advection–diffusion equations assuming a bimodal Gaussian distribution (47). From the area under the curves of the two Gaussian populations the concentrations of the bound and the free molecular chaperones were evaluated.

### Aggregation Kinetics.

The aggregation of 70 μM α-syn in the absence and presence of 0.5 μM, 1 μM, and 2 μM GFP and unlabeled/labeled αB-c in PBS, pH 7.2, with 0.1$\%\;{NaN}_{3}$ were followed by recording the increase in ThT fluorescence at 480 nm upon excitation at 440 nm. One hundred-microliter samples were incubated in a 96-well plate in a plate reader (Fluostar Optima; BMG Labtech) at 37°C. ThT concentration was 20 μM. All aggregation experiments were operated in the presence of 5% preformed second-generation fibrils (46).

### Kinetic and Thermodynamic Experiments.

Fibrils were diluted to the indicated concentrations in PBS, pH 7.2, with 0.1$\%\;{NaN}_{3}$ and incubated with 1 μM αB-c. For equilibrium experiments at 25°C, endpoint measurements were taken after 3 d. Aliquots of the chaperone–fibril system were measured on the microfluidic diffusion device at the same temperature at which they were incubated to avoid disturbing equilibrium. To minimize protein sticking to the sides of the polydimethylsiloxane devices, 0.1% Tween-20 was added to the flanking buffer streams (this is not expected to interfere with the sample, since contact between the sample and the buffer streams is minimal and short-lived).

### Diffusion Image Analysis and Fitting.

The 12 images taken along the diffusion channel were processed into a set of 12 lateral scan profiles, which was then fit to a set of simulated basis functions (24, 47). A basin-hopping algorithm with a Broyden–Fletcher–Goldfarb–Shannon minimization was used to find the linear combination of radii that gives the lowest residuals. Minimizations were run with an accuracy value (criteria for accepting a minimum) of $10^{-8}$ and epsilon value (step width of optimization) of $10^{-8}$. The fit is also penalized by a term multiplied by an empirically determined regularization coefficient α, which serves to reduce overfitting.

Each fit was run 10 to 13 times increasing α from $10^{-6}$ to $10^{-2}$ taking three steps per order of magnitude, which causes the number of radii with nonzero coefficients to decrease and the residuals to increase as the fits become more constrained (*SI Appendix*, Fig. S7*A*). While α is low the residuals remain almost constant since decreasing the number of radii fit to the experimental profiles does not dramatically impact the quality of the fit.

At some α (the value varies for each fit) there is a relatively large increase in the residuals. The magnitude of this jump reports on whether the sample is monodisperse or polydisperse (24). Empirically, if the residuals jump is smaller than $10^{-7}$ the sample is best described as monodisperse. If it is larger than $10^{-7}$ the sample is best described as polydisperse (*SI Appendix*, Fig. S7*B*). The fit immediately before the jump in residuals was taken as the best fit and used for further analysis. The tunable regularization coefficient allows us to find the simplest fit that also fully describes the data for various sets of data profiles, since the best fit differs for each.

The fitting program always includes a peak at the smallest possible radii in the range of simulated basis functions, attributing 0 to 10% of diffusion to this small radius. This artifact is due to a mismatch between the simulated diffusion profiles and the channel geometry. The artifact peak was removed by summing the two remaining populations and recalculating the percentages of free and bound chaperone.

### Native Mass Spectrometry.

Spectra were acquired on a modified quadrupole-time-of-flight (TOF) instrument (Waters Limited) following a previously described protocol (48). Samples at 20 μM were electrosprayed from gold-coated glass capillaries made in-house. The instrument parameters were as followed. αB-c wild type: source pressure 6.0 mbar, cone 50 V, extractor 3 V, collision gas Ar, collision energy 50 V, ToF pressure $3.1\times 10^{-6}$ mbar. αB-c-GFP: as above except cone 200 V, extractor 9 V, collision gas ${SF}_{6}$, collision energy 200 V. Spectra are shown with linear background subtraction and Gaussian smoothing with a window of 50 points. The spectra were fit with the UniDec software using an oligomer mass list of between 5 and 50 subunits and a peak width of 20 *m/z* units (49).

### Equilibrium and Kinetic Curve Fitting.

We fit the data with a noncooperative, single-site binding model, where free chaperones C bind to free fibril binding sites F, forming a complex CF. The equilibrium between free and bound states is governed by the forward and reverse reaction rates, which are described by rate constants $k_{on}$ and $k_{o\mathrm{ff}}$, respectively:

$$\left[C\right]+\left[F\right]\overset{k_{on}}{\underset{k_{off}}{⇌}}\left[CF\right].$$

The rate of the reaction can be expressed

$$\frac{d\left[CF\right]}{dt}=k_{on}\left[C\right]\left[F\right]-k_{o\mathrm{ff}}\left[CF\right].$$ [1]

We substitute the expressions obtained from the conservation of mass $\left[C\right]=\left[C_{t}\right]-\left[CF\right]$ and $\left[F\right]=\left[F_{t}\right]-\left[CF\right]$ into Eq. **1** to obtain a quadratic expression:

$$\frac{d\left[CF\right]}{dt}=k_{on}{\left[CF\right]}^{2}-\left(k_{on}\left(\left[C_{t}\right]+\left[F_{t}\right]\right)+k_{o\mathrm{ff}}\right)\left[CF\right]+k_{on}\left[C_{t}\right]\left[F_{t}\right],$$ [2]

where $\left[C_{t}\right]$ and $\left[F_{t}\right]$ are the total chaperone and fibril mass concentration, respectively. At equilibrium, the forward and reverse rates are equal and therefore

$$\frac{d\left[CF\right]}{dt}=0.$$ [3]

Setting Eq. **2** equal to 0, substituting the definition of the apparent dissociation constant $K_{D}=\frac{k_{off}}{k_{on}}$, and solving for the fractional occupancy $\frac{\left[CF\right]}{\left[C_{t}\right]}$, we arrive at the expression used to fit the equilibrium data:

$$\frac{\left[CF\right]}{\left[C_{t}\right]}=\frac{\left(\left[F_{t}\right]+K_{D}+\left[C_{t}\right]\right)-\sqrt{{\left(\left[F_{t}\right]+K_{D}+\left[C_{t}\right]\right)}^{2}-4\left[F_{t}\right]\left[C_{t}\right]}}{2\left[C_{t}\right]}.$$ [4]

To fit the kinetic data, we solve Eq. **2** by the method of partial fractions to find

$$\frac{\left[CF\right]}{\left[C_{t}\right]}=\left[F_{t}\right]\frac{1-e^{k_{on}\left(x_{+}-x_{-}\right)t}}{x_{-}-x_{+}e^{k_{on}\left(x_{+}-x_{-}\right)t}}=\frac{1}{\left[C_{t}\right]}\frac{1-e^{k_{on}\left(x_{+}-x_{-}\right)t}}{x_{+}^{-1}-x_{-}^{-1}e^{k_{on}\left(x_{+}-x_{-}\right)t}},$$ [5]

where

$$x_{\pm }=\frac{k_{on}\left(\left[C_{t}\right]+\left[F_{t}\right]\right)+k_{o\mathrm{ff}}\pm \sqrt{k_{on}^{2}{\left(\left[C_{t}\right]-\left[F_{t}\right]\right)}^{2}+k_{o\mathrm{ff}}^{2}+2k_{on}k_{o\mathrm{ff}}\left(\left[C_{t}\right]+\left[F_{t}\right]\right)}}{2k_{on}}.$$ [6]

In this expression, we constrain the value of $\left[C_{t}\right]$ and take the following terms as fitting parameters: $k_{on}$, $k_{o\mathrm{ff}}$, and $\left[F_{t}\right]$, as we do not know the actual number of fibril binding sites.

In the limit where time recovers infinity, Eq. **5** goes to the equilibrium solution (Eq. **4**).

### Curve Fitting to Obtain Thermodynamic and Kinetic Parameters.

The van’t Hoff equation gives the relationship between the equilibrium constant $K_{eq}$ (related to the apparent dissociation constant by $K_{eq}=1/K_{D}$) and temperature T:

$$\frac{d\ln K_{eq}}{d\left(1/T\right)}=-\frac{\Delta H}{R},$$ [7]

where ΔH is the enthalpy and *R* is the gas constant. We arrive at the linear form of the van’t Hoff equation, using $K_{D}$ instead of $K_{eq}$ by combining Eq. **7** with the relationship between Gibbs free energy and the equilibrium constant $\Delta G=-RT\ln K_{eq}$, and the definition of Gibbs free energy ΔG=ΔH−TΔS:

$$\ln\;K_{D,app}=\frac{\Delta H}{RT}-\frac{\Delta S}{R}.$$ [8]

By adding the contribution of the change in heat capacity $\Delta C_{p}$ to the enthalpy and entropy (Eqs. **9** and **10**),

$$\Delta H=\Delta H_{Tref}+\Delta C_{p}\left(T-T_{ref}\right)$$ [9]

$$\Delta S=\Delta S_{Tref}+\Delta C_{p}\left(\ln\;T-\ln\;T_{ref}\right),$$ [10]

we derive

$$\ln\;K_{D,app}=\frac{\Delta H_{Tref}}{RT}-\frac{\Delta S_{Tref}}{R}+\frac{\Delta C_{p}}{R}\left(1-\frac{T_{ref}}{T}\right)-\frac{\Delta C_{p}}{R}\ln\left(\frac{T}{T_{ref}}\right),$$ [11]

where $\Delta H_{Tref}$ and $\Delta S_{Tref}$ are the enthalpy and entropy changes at a reference temperature. The reference temperature $T_{ref}$ is chosen to be 310 K.

We also estimate the free energy barrier of the binding reaction from this dataset by constructing a model which combines polymer theory and Kramer’s problem of escape from a metastable state considering the change in association rate constant $k_{on}$ of αB-c binding to the fibrils (Φ) as a function of temperature (32):

$$k_{on}=1,000\cdot D\cdot N_{A}\cdot r_{e\mathrm{ff}}\cdot e^{-\beta \Delta G^{‡}}=1,000\cdot D\cdot N_{A}\cdot r_{e\mathrm{ff}}\cdot e^{-\beta \Delta H^{‡}}e^{\beta T\Delta S^{‡}}.$$ [12]

In order to respect the contribution of the heat capacity change to the activated state $\Delta C_{p}^{‡}$ we combined Eqs. **9**, **10**, and **12** to

$$\begin{array}{ll} k_{on} & =1,000\cdot D\cdot N_{A}\cdot r_{e\mathrm{ff}}\cdot e^{-\beta \left[\Delta H_{Tref}^{‡}+\Delta C_{p}^{‡}\left(T-T_{ref}\right)\right]}\\ & \;\times e^{\beta \left[T\Delta S_{Tref}^{‡}+\Delta C_{p}^{‡}\left(\ln\;T-\ln\;T_{ref}\right)\right]}, \end{array}$$ [13]

where *D* is the diffusion constant of a segment of the protein (comprising three amino acids, the Kuhn length of a polypeptide chain) and has a numerical value of approximately $5\times 10^{-10}m^{2}s^{-1}$, $N_{A}$ is the Avogadro constant, and $r_{e\mathrm{ff}}$ is the characteristic distance that can be computed from

$$r_{e\mathrm{ff}}=\frac{b_{0}}{\pi \sqrt{2n}}$$ [14]

With *b*_0_ being around 1 nm, the Kuhn length of a polypeptide chain, and *n* the number of protein residues.

## Supplementary Material

Supplementary File

## Data Availability

All study data are included in the article and/or supporting information.
