# Supplementary material for: The binding of the small heat-shock protein αB-crystallin to fibrils of α-synuclein is driven by entropic forces
Source: Proc Natl Acad Sci U S A. 2021 Sep 13;118(38):e2108790118. doi: 10.1073/pnas.2108790118 (PMC8463877; doi:10.1073/pnas.2108790118)
Supplement: Supplementary File [file pnas.2108790118.sapp.pdf]

The binding of the small heat-shock protein αB-crystallin to fibrils of α-synuclein is driven by entropic forces

Scheidt, Carozza et al. 10.1073/pnas.XXXXXXXXXX

Supporting Information (SI)

Table S1. Summary of the kinetic parameters k_{on} and k_{off} and the dissociation constants K_d for the binding of αB-c to α-syn fibrils at different temperatures. Values were obtained by fitting the kinetic data to Equation 6 (Materials and Methods). Note that at 37°C where the binding curve reaches saturation, the value for k_{off} and therefore also the value of K_d obtained from the fit is subjected to be more error prone than the other dissociation constants at lower temperatures. The fit still gives us an estimate of the binding affinity, and still reports well on k_{on}.

	αB-c		
T (°C)	k_{on} ($M^{-1}s^{-1}$)	k_{off} (s^{-1})	K_d (nM)
7	0.6 ± 0.4	$2.6\times10^{-5} \pm 1.6\times10^{-5}$	45310 ± 15890
10	0.3 ± 0.1	$4.2\times10^{-6} \pm 6.3\times10^{-7}$	12330 ± 4465
17	0.7 ± 0.3	$4.1\times10^{-6} \pm 1.9\times10^{-6}$	6118 ± 2722
20	2.4 ± 0.9	$9.8\times10^{-6} \pm 2.8\times10^{-6}$	4075 ± 1701
25	7.1 ± 2.6	$2.7\times10^{-6} \pm 8.6\times10^{-7}$	373 ± 238
30	31.4 ± 12.9	$1.8\times10^{-6} \pm 1.1\times10^{-6}$	58 ± 53
37	199.5 ± 86.8	$1.2\times10^{-16}\,^{+1.9\times10^{-6}}_{-1.2\times10^{-16}}$	≤ 10

1. Marty MT, et al. (2015) Bayesian Deconvolution of Mass and Ion Mobility Spectra: From Binary Interactions to Polydisperse Ensembles. *Anal. Chem.* 87(8):4370–4376.

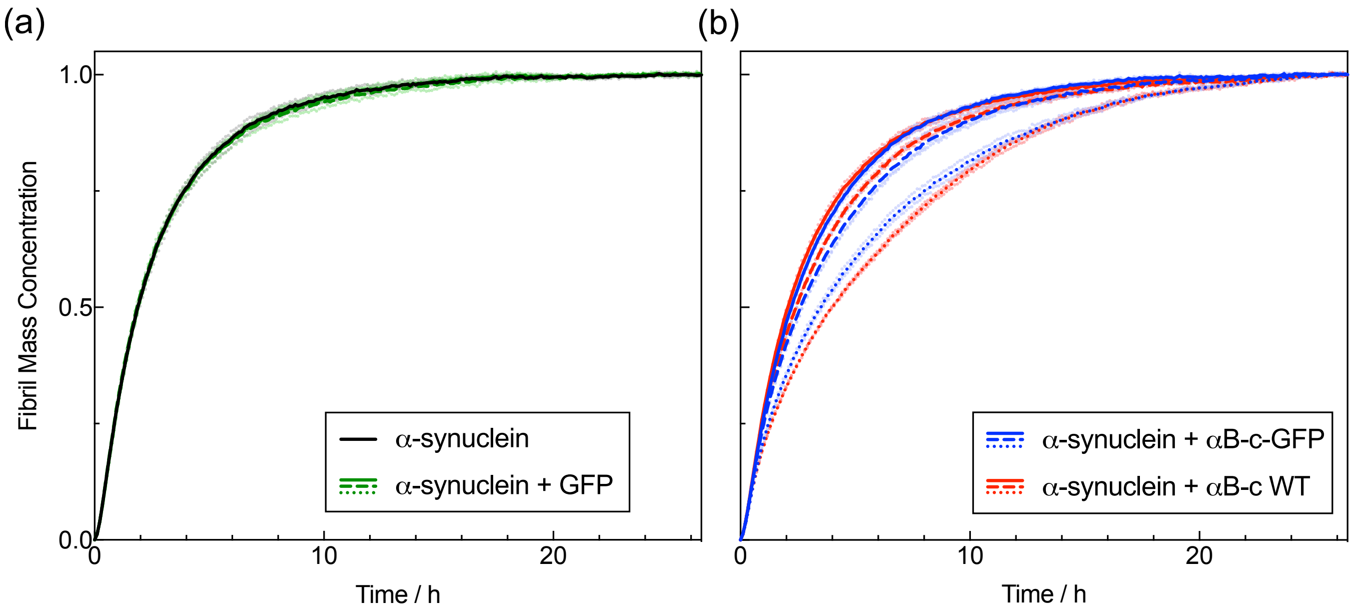

Fig. S1. Kinetic reaction profiles of 70 μM α-syn solutions are shown (a) in the absence (black curve) or in the presence of either 0.5 μM (solid curve), 1 μM (dashed curve) and 2 μM (dotted curve) of GFP (green curves) or (b) unlabelled/labelled αB-c including 5% seeds in PBS, pH 7.2 with 0.1% NaN_3 (red and blue, respectively). Each curve is an average of three independent measurements with individual measured points shown in each plot (faded colors).

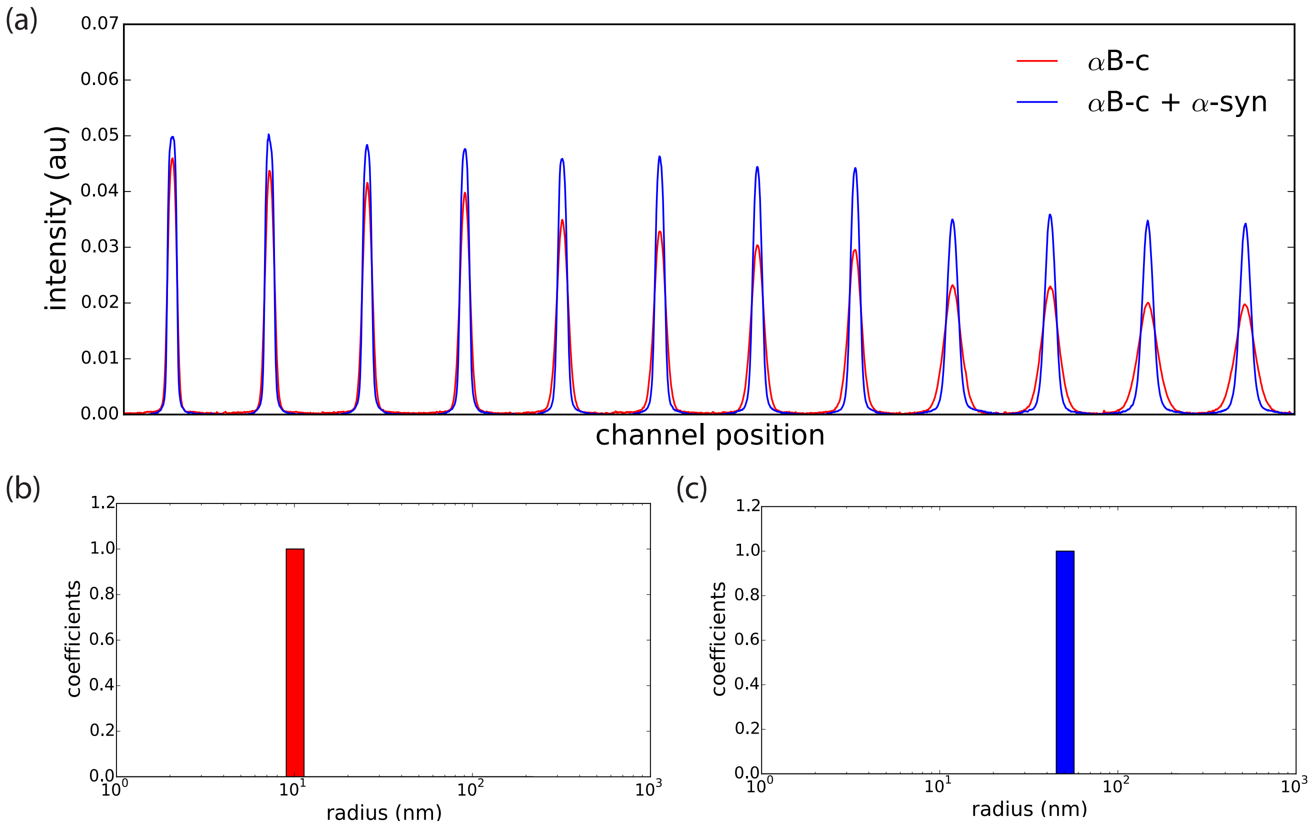

Fig. S2. (a) Complete set of 12 diffusion profiles for αB-c alone and αB-c incubated with α-syn fibrils and exhibiting 100% binding. Histograms of the sample size distribution for (b) αB-c alone and (c) αB-c with α-syn fibrils.

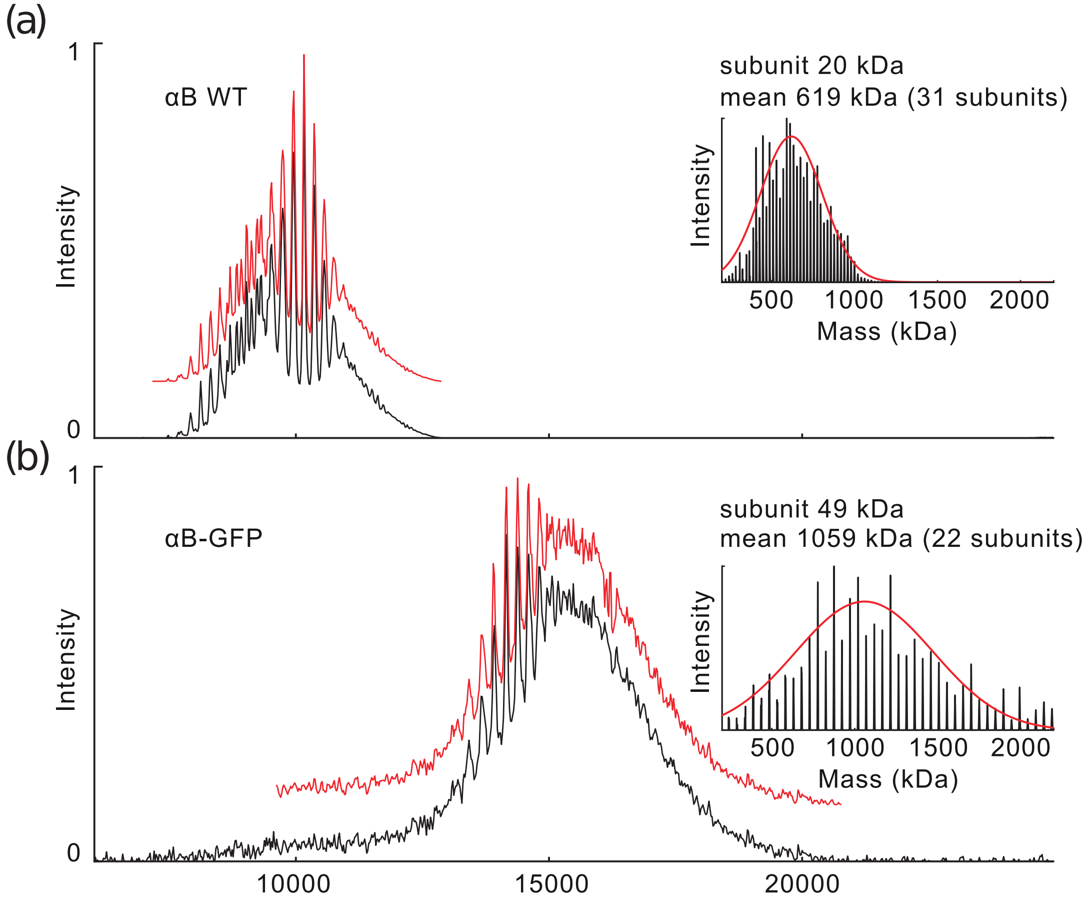

Fig. S3. Native mass spectrometry of (a) wild-type αB-c and (b) αB-c-GFP fusion. The mass spectra are shown in black with a Bayesian deconvolution fit in red(1). Charge-deconvolved mass distributions underlying the fit are shown as insets. The mean and standard deviation for the fit mass distributions was 619 ± 187 kDa $(31.0 \pm 9.3$ subunits) for αB-c WT and 1059 ± 417 kDa $(21.6 \pm 8.5$ subunits) for αB-c-GFP. Please note that, at this mass spectral resolution, the fit enables only an estimate of the average mass distribution and detailed interpretation of relative intensities of the different oligomers is not appropriate.

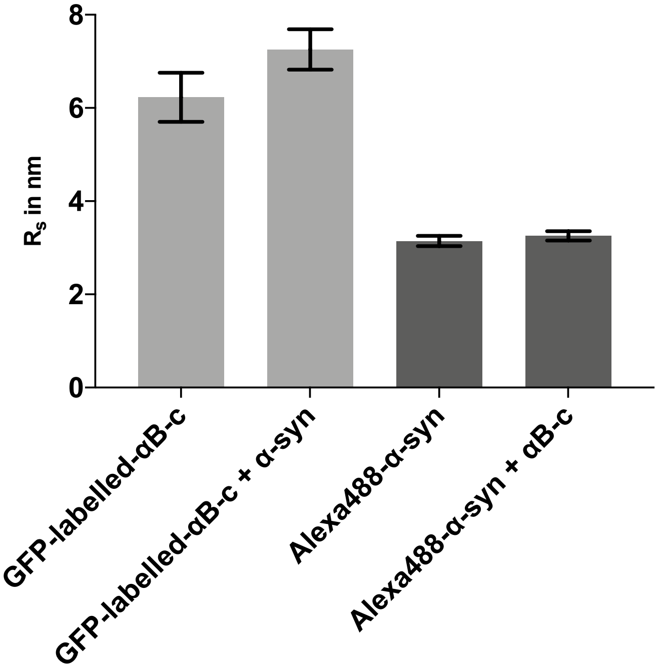

Fig. S4. The binding of 1 μM αB-crystallin and 10 μM monomeric α-synuclein, labelled with GFP or Alexa488, respectively, was investigated with diffusional sizing at 23°C. The hydrodynamic radius shows no significant change in the presence of both monomeric α-syn and αB-c compared to their individual size, thus indicating no specific binding between both species.

Scheidt, Carozza et al. 10.1073/pnas.XXXXXXXXXX

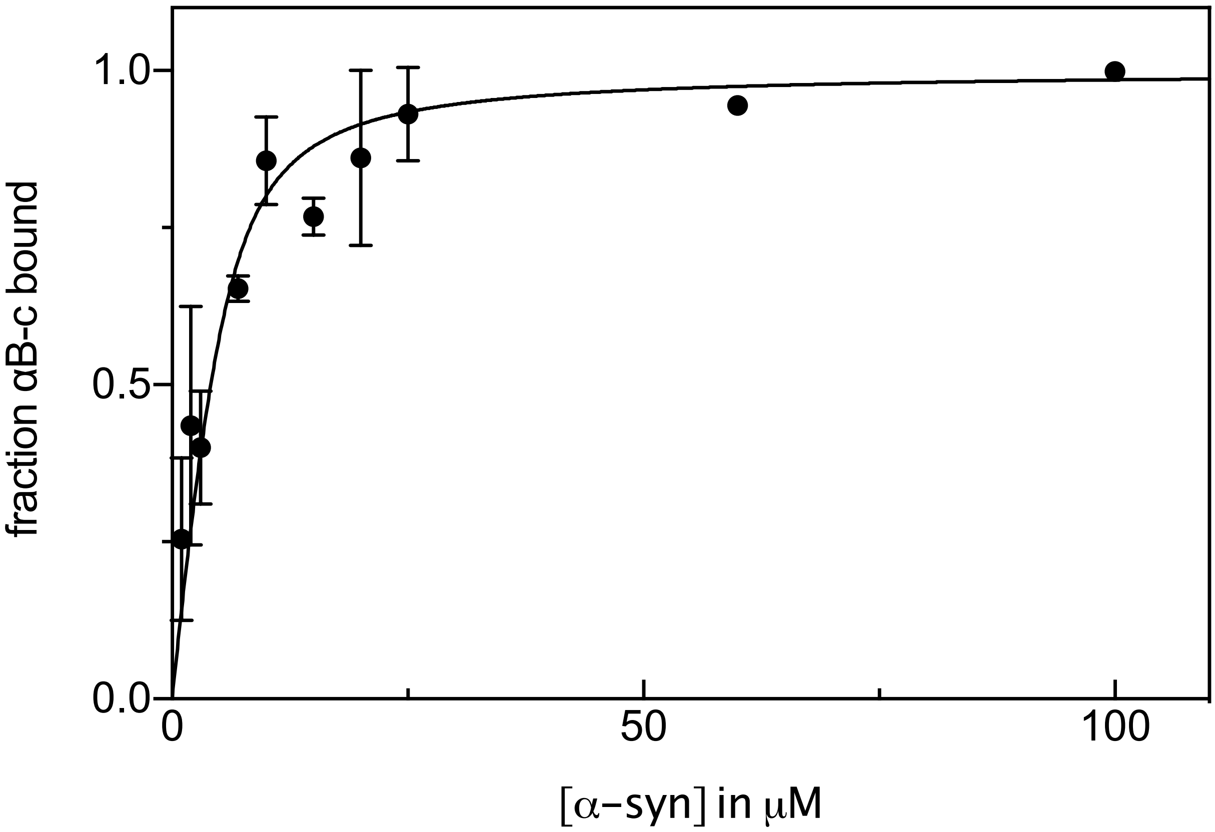

Fig. S5. The chaperone αB-c binds to α-syn fibrils in the high nanomolar affinity range $\mathrm{K}_{\mathrm{D},25°\mathrm{C}} = 261 \pm 76$ nM. The concentration of αB-c was kept at 1 μM and the concentration of α-syn fibrils was varied over two orders of magnitude to obtain a titration curve, and the data were fit according to a single-site binding model, with an α-syn binding site ratio of 0.186 binding sites per α-syn monomer. Error bars represent standard deviations from at least two independent measurements.

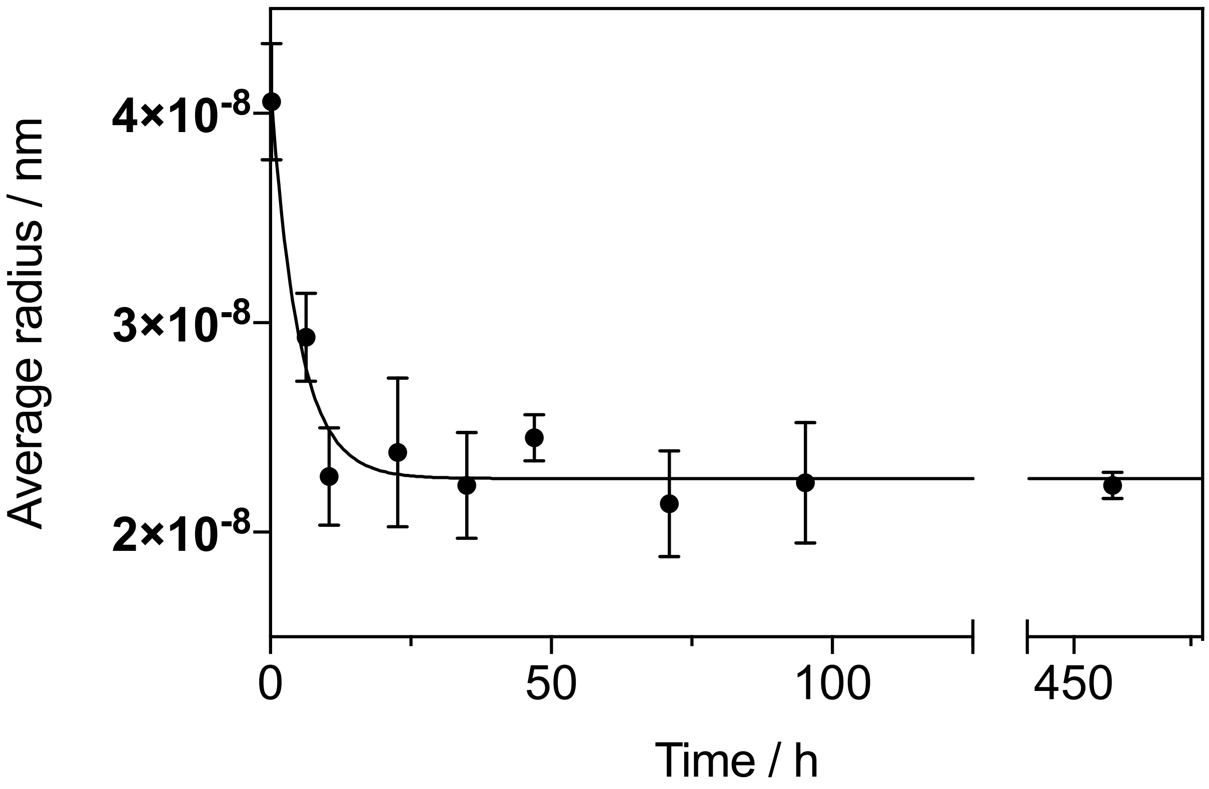

Fig. S6. Dissociation kinetic of αB-c (1 μM) in a saturated α-syn fibril solution at $7^{\circ}\mathrm{C}$. The data was fitted to a one phase exponential decay equation and the rate constant is $\mathrm{k_{off,7^{\circ}C}} = 5.6 \times 10^{-5} \pm 1.2 \times 10^{-5}\ \mathrm{s^{-1}}$.

 Scheidt, Carozza et al. 10.1073/pnas.XXXXXXXXXX

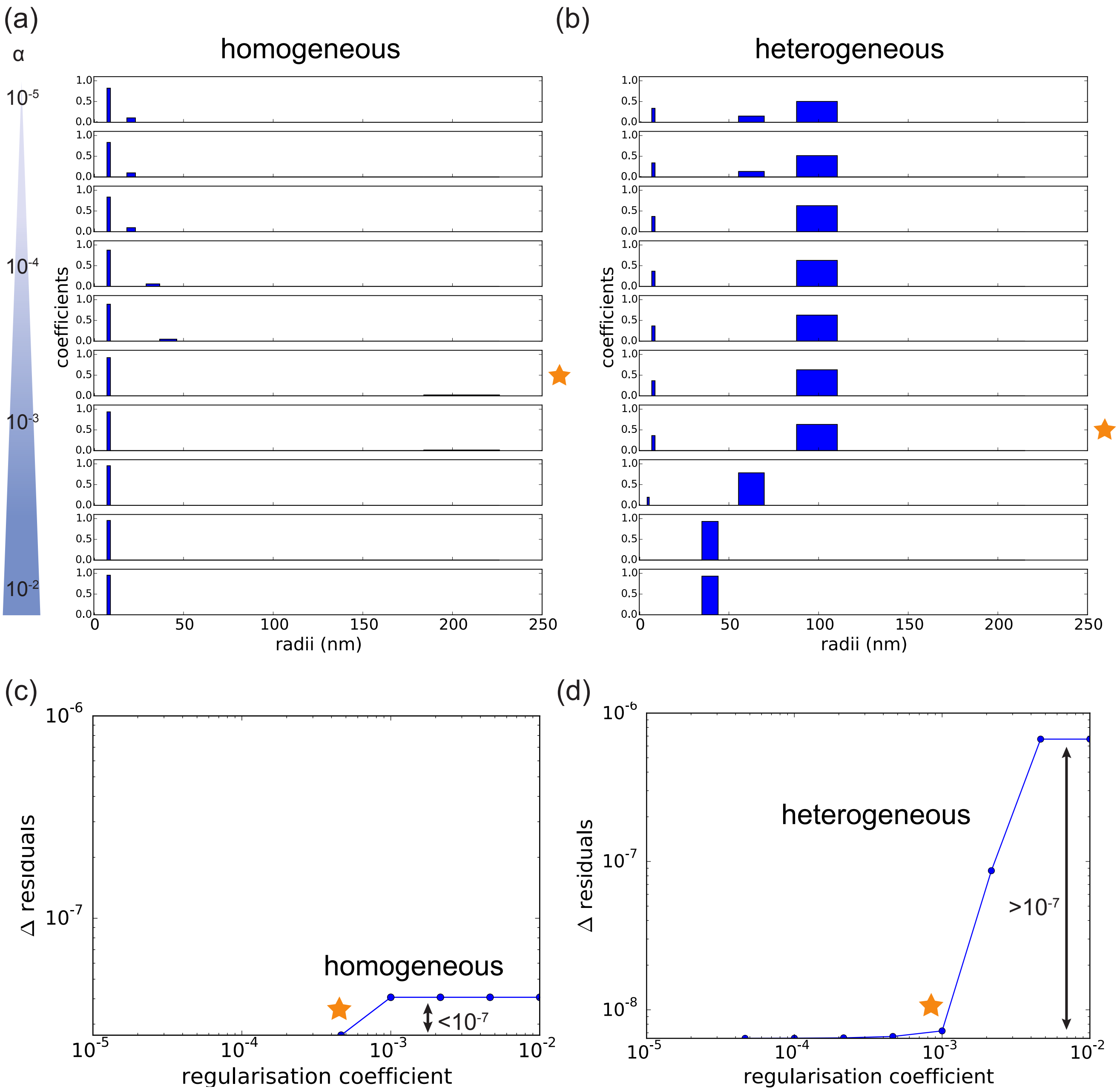

Fig. S7. Representative fits for (a) homogeneous (αB-c alone) and (b) heterogeneous compositions (αB-c incubated with fibrils), exhibiting binding. Higher regularization coefficients penalize the number of individual components considered in the fitting. The change in the residuals relative to the sum of the residuals of the first fit ($\alpha = 10^{-5}$) for the homogeneous (c) and heterogeneous (d) samples plotted against α demonstrate that there is some critical value of α, at which point there is a jump and the experimental profiles are no longer well described by the fit. The magnitude of that jump reports on whether the samples are homogeneous or heterogeneous (the cutoff value is $\alpha \approx 10^{-7}$). The fit immediately before the jump in residuals (marked with a star) is taken as the best fit for that set of profiles.
